# This is me: A qualitative investigation of young people’s experience of growing up with visual impairment

**DOI:** 10.1371/journal.pone.0254009

**Published:** 2021-07-07

**Authors:** Alexandra O. Robertson, Valerija Tadić, Jugnoo S. Rahi

**Affiliations:** 1 Population, Policy and Practice Research & Teaching Department, UCL Great Ormond Street Institute of Child Health, London, United Kingdom; 2 Great Ormond Street Hospital NHS Foundation Trust, London, United Kingdom; 3 National Institute for Health Research (NIHR) Biomedical Research Centre at Moorfields Eye Hospital NHS Foundation Trust and UCL Institute of Ophthalmology, London, United Kingdom; 4 Ulverscroft Vision Research Group, London, United Kingdom; Centre for Sexual Health & HIV/AIDS Research, ZIMBABWE

## Abstract

**Background:**

Childhood visual impairment (VI) has a profound impact on many aspects of childhood and adolescence. This is well-documented in cross-sectional and/or quantitative studies utilizing self-report instruments which compare children with and without VI. Young people’s views on the experience of growing up with VI as a developmental, change-driven process remain largely unexplored.

**Methods:**

As part of our broader research programme on quality of life of visually impaired children and young people in the United Kingdom, in-depth, semi-structured interviews were conducted between March and June 2015, with a stratified sample of 17 young people with VI, aged 16–19 years. An age-sensitive, empirically-based topic guide encouraged retrospective reflections on participants’ experiences of growing up with VI, including age-normative and vision-specific challenges.

**Results:**

Descriptions of growing up with VI largely centered on an overarching higher-order theme labelled *becoming me*. Four themes representing everyday activities, attitudes, preferences and perceptions in relation to i) *social relationships*, ii) *independence and responsibilities*, iii) *the future*, and iv) *rising to challenges* emerged and were used by participants in their description of three stages in which they developed a sense of self: i) *laying the foundations*, ii) *testing the waters*, and iii) *this is me*. Differences in manifestation of VI influenced how young people made sense of their experiences and their sense of self.

**Conclusions:**

Findings are discussed in relation to normative and vision-specific changes in psychosocial development during adolescence, including the development of identity. They highlight the need for ongoing monitoring of subjective well-being in a clinical population with a unique early life course trajectory.

## Introduction

Childhood is a transient period of life course, characterized predominantly by periods of growth and change, be it at the level of sexual maturity, specialized neurons in the brain, developmental capacity or emotional understanding. The subjective experience of some elements of growth or change during childhood is well-documented. For example, adolescence, the pinnacle of childhood, has traditionally been viewed as a period of *stress and storm* [1], with changing hormones triggering negative emotions, unpredictable behaviour and mood swings [2, 3]. Recent, advanced neuroimaging techniques have shed light into these behavioural and psychological upheavals, demonstrating the role of functional changes in the brain [4]. It is generally agreed that adolescence constitutes a vulnerable period in which morbidity and susceptibility to mental health conditions, such as anxiety and depression can increase [5].

When a child is living with a disability, the subjective experience of childhood and adolescence is likely to be qualitatively different from that of a child living without a disability. In addition to navigating normative, age-specific changes and challenges, children and young people living with disability will experience far-reaching, complex and dynamic issues associated specifically with their condition.

Currently, in the UK, 2 in every 1000 children and young people are estimated to be visually impaired or blind. Most will have been born with visual impairment or develop this in early life and have therefore grown up without normal vision [6, 7]. Existing qualitative studies have described the profound impact of VI during childhood and adolescence, showing that children and young people living with VI experience challenges participating in age-appropriate activities, such as reading, watching live sports or concerts, or playing outdoor games, and are subsequently unable to pursue the activities they enjoy the most [8, 9]. A robust evidence base documents the impact of VI on aspects of cognitive [10, 11], social [12, 13], and sensorimotor [14] development, demonstrating developmental delay among those diagnosed with VI early during childhood [15].

There is some literature which has focused specifically on psychological well-being among individuals who have grown up with VI, however conclusions about the impact of VI during childhood and adolescence are mixed. For example, remarkable similarities between visually impaired individuals and their sighted peers in psychosocial well-being, incidence of depression, and aspects of self-concept are documented [16–20]. Alternatively, adolescents living with VI have been shown to have smaller social networks [16, 21, 22], and score higher in assessments of obsession-compulsion, hostility, and paranoid ideation than their sighted counterparts [23].

Existing studies exploring the impact of VI on aspects of psychological and emotional well-being during childhood and adolescence largely rely on data collected using self-report measures, which are administered at one time point [24, 25]. These tools afford an optimal, quantitative approach for comparing different groups, but offer limited insight into the lived experience of being visually impaired, and of how young visually impaired people make sense of their experience of growing up with VI. Qualitative research studies are scarce and, where they do exist, have explored children and young people’s current experiences, primarily in relation to restricted daily activities [8].

As part of our broader research programme on quality of life of visually impaired children and young people [26, 27], we interviewed young people living with VI about their daily lives with an aim to understand more about their experiences living with VI. In conducting in-depth, semi-structured interviews with young people, we took the opportunity to capture, and present in this paper, young people’s *retrospective* experience of growing up with VI. Specifically, we explored the research questions: “How do young people describe their experience of growing up with VI?” and “What are the most important issues related to growing up with VI?”

## Materials and methods

### Sample

Young people were eligible if they were *i*) visually impaired, severely visually impaired or blind (visual acuity in the *better eye* LogMAR 0.48 or worse, or Snellen worse than 6/18, or additional visual defects causing VI [28]) due to any visual disorder, but without any other significant impairment (i.e. learning, sensory or motor); and *ii*) aged 16–19 years. We used a random stratified sampling approach to select participants from 2 patient populations comprising those attending the Department of Ophthalmology at Great Ormond Street Hospital and the Paediatric Glaucoma Service and Genetic Eye Disease Service at Moorfields Eye Hospital, London, United Kingdom (UK). Participants were approached by postal invitation to take part in semi-structured interviews as the first part of our broader research programme exploring vision-related quality of life and functional vision [26, 27].

### Procedure

In-depth, semi-structured interviews were conducted at participants’ homes, between March and June 2015 by a researcher (AR) with robust training and experience in using qualitative methods and conducting in-depth interviews. An interview topic guide was developed *de novo* (based on a literature review of subjective experience of a chronic condition/disability during childhood, with specific focus on VI) to explore many areas of everyday life (e.g. home, school, and leisure). Probes, such as ‘What changed for you?’, ‘How has that changed over time/as you got older?’, and ‘Can you remember when things were different?’ encouraged a retrospective account of the experience of growing up with VI (S1 File). Participants were encouraged to talk to the interviewer independently (without a parent present) and an ice-breaker activity preceded each interview. The number of interviews conducted (sample size) was determined using the principle of data saturation [29, 30] in the context of the broader research programme. All participants received a £20 gift voucher after completing the interview. This monetary reward was not an incentive for participation.

### Qualitative data analysis

All interviews were recorded, transcribed verbatim and anonymised. Transcribed data were exported into NVivo 10 [31]. Qualitative thematic analysis was performed inductively to identify key themes spontaneously emerging from the data [32]. Analysis comprised an ongoing, circular process of open-coding completed by two researchers (AR and VT). Researchers began by coding a sample (20%) of full interview transcripts independently, before meeting to discuss the codes used, with reference to the data. Any discrepancies in the coding process were resolved through discussion and further reference to the data (both specific extracts and full transcripts). Researchers developed a code book which was used by one researcher (AR) to code the entire dataset. Rigorous and consistent coding was ensured through regular meetings between researchers to discuss any disagreements/discrepancies and further modifications to the code book. Following the initial coding of all data, data were grouped by specific codes which were reviewed for emerging themes. Two researchers (AR and VT) met to develop, label and describe higher-order themes emerging from grouped data.

### Ethical considerations

This study was approved by the National Health Service (NHS) Research Ethics Committee for Essex and East of England, UK (12-EE-0455) and adhered to the tenets of the Declaration of Helsinki. All participants and their parents gave informed written and verbal consent to participate.

## Results

Table 1 shows participants’ demographic and clinical characteristics, illustrating a wide range of characteristics with respect to the overall UK population of children and young people with VI (given the exclusion of participants with any other significant impairment) [6]. A total of 17 young people took part (40.48% participation rate).

**Table 1:** Demographic and clinical characteristics of participants.

Characteristic	*n* (%)
**Age**	
16	7 (41.18)
17	8 (47.06)
18	1 (5.88)
19	1 (5.88)
**Gender**	
Male	10 (58.82)
Female	7 (41.18)
**Ethnicity**	
White UK majority (White British)	8 (47.06)
White other (e.g. African, Polish, Turkish)	3 (17.65)
Black (British, African, Caribbean)	-
Asian (Indian, Bangladeshi, Pakistani)	4 (23.53)
Asian other (Arabic)	1 (5.88)
Chinese	-
Mixed	1 (5.88)
Severity of visual impairment*	
Low Vision: logMAR ≤ 0.46	1 (5.88)
Moderate VI: logMAR 0.48–1.00	11 (64.71)
Severe VI (SVI): logMAR 1.02–1.30	2 (11.76)
Blind (BL): logMAR ≥1.32	3 (17.65)
**Timing of onset of visual impairment**	
Early (≤2 years)	15 (88.24)
Late	2 (11.76)
**Nature of deterioration of visual impairment**	
Stable	12 (70.59)
Progressive	5 (29.41)
**Index of multiple deprivation (based on UK postal code) quintile rank [33]**	
1: most deprived	1 (5.88)
2	2 (11.76)
3	4 (23.53)
4	8 (47.06)
5: least deprived	2 (11.76)

* Severity of visual impairment categorized according to the WHO criteria [28].

Duration of in-depth interviews ranged from 40–139 minutes (Median = 88 minutes, IQR = 39). Sixteen interviews were conducted at participants’ family homes. One was conducted at the primary recruitment site. One or more parents/caregivers were present for part, or all of 4 interviews.

### Overview of themes

**Fig 1:**
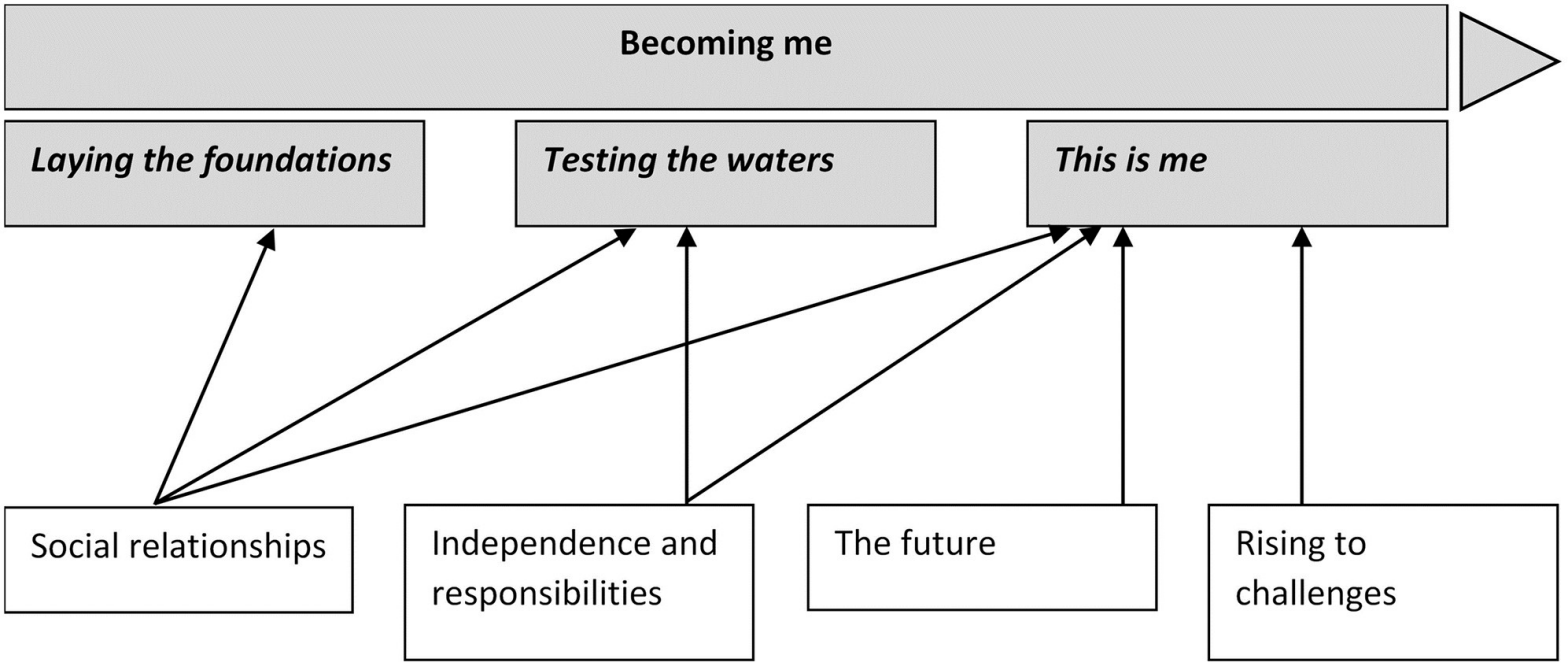
Thematic map. Shaded boxes denote higher-order themes (higher level of abstraction than the original emergent themes). White boxes present themes.

Four basic themes emerged directly from the data, representing young people’s descriptions of everyday activities and attitudes, preferences and perceptions in relation to i) *social relationships*, ii) *independence and responsibilities*, iii) *the future*, and iv) *rising to challenges*. The issues discussed within these themes are best described using an overarching higher-order theme labelled as *becoming me*, describing a process in which participants developed a sense of self during childhood and adolescence, in the context of normative aspects of everyday life, as well as vision-specific experiences. Participants described the process of *becoming me* during childhood and adolescence, with three distinct stages: i) *laying the foundations*, ii) *testing the waters* and, iii) *this is me*. As shown in Fig 1, the 4 basic themes cumulatively mapped onto these stages (i.e. the final stage incorporating elements of all 4 basic themes). Variation emerged in the experiences of those with varying manifestations of VI (i.e. in relation to the timing of onset of VI, severity of VI, and rate of deterioration of vision).

### Laying the foundations

Social relationships emerged as the single, and most important factor contributing to the first stage described by participants as important in the process of *becoming me*. During early childhood, relationships were established within family communities. Siblings and cousins were described as key playmates, and valuable friendships were established with others who attended the same nurseries and pre-schools. Participants who experienced VI from an early age (i.e. early onset ≤ 2 years) recalled their first awareness of the qualities distinguishing “*true friends*” (i.e. those who were able to accept or adapt to participants’ limited functional vision), from others who would treat them as inferior or exclude them from activities.

*“He’s grown up with me*, *so he’s seen what I can and can’t do*. *He treats me the same as he might treat anyone else*. *I’m not treated as someone who’s blind.”*

*(Male*, *moderate VI*, *early onset)*.

Regardless of manifestation of VI, true friends were described as those who could offer functional support in a way which is both appropriate and subtle, and seemingly helped *lay the foundations* for participants to develop a sense of self. Participants described their emerging confidence to participate in broader, social, public settings outside their immediate family and friendship groups.

*“[If I can’t see the board] I just put my pen down and wait*. *I’ll give my friend ‘the look’ and he’ll be like*, *yeah [and help me]”*

*(Male*, *moderate VI*, *early onset)*.

#### Differences between those with early (≤ 2 years) and late-onset VI

Social relationships and a surrounding social environment were discussed in relation to participants’ acceptance of VI, and to what extent they were able to integrate it into their sense of self. Some participants with early onset VI (≤2 years) compared themselves to their peers, describing VI as a personal attribute, which made them feel unique and interesting. From this perspective, some young people were able to seek benefits from their early diagnoses.

*“It’s never really bothered me*, *cos I’ve never seen properly*. *It’s just who I am”*

*(Male*, *SVI/BL*, *early onset)*.

Alternatively, and as stark contrast, participants with subsequent prior experience of full functional vision recalled a complex transition in their feelings of belonging and self-acceptance, occurring in the early stages of visual deterioration, which was particularly prominent when navigating social encounters. Expectations and views which had previously contributed to a sense of self had to be recalibrated.

*“You don’t want family or friends who you’ve seen before*, *and have seen you ‘seeing’*, *noticing that it has got bad*.*”*

*(Male*, *SVI/BL*, *late onset)*.

*“It really upset me*. *I lost so many friends because I wasn’t Pearl*. *I was Pearl who just found out she has got an eye disability*. *So I had to mature like that [snaps fingers]*. *I had to do things for myself […] A lot of people thought I had changed […]”*

*(Female*, *moderate VI*, *late onset)*.

*“My eyes had got worse*. *And then I had to adapt myself again […] So it’s like losing someone*. *Cos you’re constantly having to*, *like*, *grieving I suppose*.*”*

*(Female*, *SVI/BL*, *late onset)*.

### Testing the waters

Participants recalled emerging and developing autonomy during childhood, initially afforded by parents and caregivers who gradually permitted, or encouraged independence through activities such as visiting local cafes or shops alone. Greater independence and autonomy, granted by parents, meant that young people could *test the waters* of a broader physical and social environment extending beyond their immediate home or school life. In doing so, participants described their developing confidence. New strategies were established for overcoming vision-specific challenges in the absence of functional support from parents.

*“At the end of primary school I started going out by myself*. *It was just going into town*, *in a massive group [of friends]*. *Then I started going places by myself*, *mostly to go and meet people”*

*(Male*, *moderate VI*, *early onset)*.

*“Only when I started going out*, *I realized I can see the board [timetable at the station] a lot better if I take a photo of it with my phone*. *I rely on my phone”*

*(Female*, *moderate VI*, *early onset)*.

Exposure to new surroundings, however, increased the potential for negative experiences in public settings, which were described by some participants in the form of humiliation or frustration as a result of other people not understanding the impact of VI, and in turn influenced participants’ sense of self in a new, broader social context.

*“Sometimes people are really arsy towards you*. *Me and my friend went to the cafe and we couldn’t see the menu*, *so we asked “What sandwiches have you got*?*”*, *and the lady said “Can’t you see*?!*” Sometimes people don’t react well*. *We [VI persons] are like aliens to them*.*”*

*(Female*, *moderate VI*, *early onset)*.

Perhaps as a result of these negative experiences, some participants described an emerging attitude towards using assistive devices, such as magnifiers, or white canes when in public. One participant described a time when he would “*forget*” to take a cane with him when he left home, or “*lose”* his cane at home. Whilst this strategy alleviated some of the emotional distress caused by using assistive devices in public, it introduced a new challenge of *testing the waters* without any support, and tripping or falling over was often a consequence.

*“I used a cane when I was younger, but now I’m reluctant to take it out. I don’t want to be singled out. There was one day when I tried [to read the menu] and somebody laughed at me.”*

*(Male, SVI/BL, early onset)*.

*“I don’t like using my [cane] in front of my family*. *I will only use it if I’m alone and I really have to use it*.*”*

*(Male*, *SVI/BL*, *early onset)*.

For many participants, expanding social environments were associated with increasing awareness of age-appropriate, normative activities, and specifically, realizations of the discrepancy between those which participants were capable of, and those which might be possible if participants had better functional vision. The majority of participants described the inability to drive, or take driving lessons, for example, as an increasingly salient barrier towards independence, which had profound emotional consequences. Feelings of frustration, annoyance and despair intensified when participants reached an age in which they were legally eligible to drive, and witnessed their peers learning to drive.

*“[Driving] is something which has been building up in me over time and it didn’t really affect me till I reached the age of 17 […] My sister passed her test the year before and at that point I wasn’t worried*. *But when my friends started to take it and pass it*, *it really hit me and I realized that I definitely wouldn’t be able to do that*. *Before it always seemed like something that was gonna happen in the future*.*”*

*(Male*, *moderate VI*, *early onset)*.

#### Differences based on severity of VI

Being on the threshold of participation in age-appropriate activities, such as driving, was at times associated with greater, intensified negative emotions than being unable to engage with the activity outright, distinguishing participants with milder forms of VI from those with severe VI.

*“I’d rather be able to do it [drive] but not do it*, *than not be able to do it and want to do it*. *[…] It’s about having that choice*. *But I’ll never have it*, *so*, *just got to deal with it*.*”*

*(Female*, *moderate VI*, *early onset)*.

*“That little niggle in the back of your mind ‘oh what happens if my eyesight gets better*?*’ I tried to push myself to see a number plate and it wasn’t working*. *I tried my damned hardest to see it*.*”*

*(Female*, *moderate VI*, *early onset)*.

*“I’ve always known I won’t be able to drive*. *Can you imagine*, *a blind person driving*! *[laughs]”*

*(Male*, *SVI/BL*, *early onset)*

### This is me

The final stage described by participants as important to the process of *becoming me* incorporated changes in social relationships, the nature of independence and responsibilities, and for the first time, thoughts, perceptions, and activities related to the future. Participants described accumulating normative aspects of childhood and adolescence, and vision-specific challenges, resulting in realizations of i) who they had become, and ii) who they would be in the future.

During this later stage, adults such as teachers and parents were increasingly respected and appreciated as equals versus superiors or carers. Some participants identified teachers who they “*could have a laugh with”*, or whom they identified as “*mates*”.

*“[On a school trip] Instead of giving me a learning support assistant*, *they [the school] actually paid for a ticket for Mr J to go*. *He’s just like one of the lads*!*”*

*(Male*, *SVI/BL*, *early onset)*.

These adults were valued as role models, and contributed to participants’ developing sense of self, providing a sense of direction in relation to future ambitions and, in particular, the type of career or activity which would be possible in light of VI.

*“My uncle’s a lawyer*. *And he’s told me stories about people he’s dealt with […] I like the idea of it and how*, *even though they’ve had such a rough background*, *he’s helped them*. *I would like to do that for other people as well”*

*(Male*, *SVI/BL*, *early onset)*.

Parents played an important role as advocates for future plans which are realistic, providing guidance, giving suggestions, and ensuring young people developed necessary independent-living skills. Input from parents, however, could be perceived as overprotective, and young people described their frustrations about imposed limitations.

*“I want to go and study abroad for a year*. *I want to go to China but my mum won’t let me go*. *But I want to do it all […] I wanted to study in America*, *but again*, *my mum*, *she’s really iffy about that*.*”*

*(Female*, *moderate VI*, *early onset)*.

Newly established romantic relationships emerged in participants’ discussions of adolescence, and were described as enjoyable and comforting, and favoured in light of having somebody other than a parent, carer or teacher to offer functional support. These types of relationships triggered thoughts about the future, specifically about moving away from home, and having a family.

*“He [my boyfriend] helps me*. *Say*, *if we’re out at a restaurant*, *he reads the menu for me and things like that*.*”*

*(Female*, *SVI/BL*, *late onset)*.

*“I’m guessing*, *as you grow up*, *being an adult*, *you just want to be on your own*, *or with your partner*. *And then start a family*. *That’s what I want*.*”*

*(Female*, *moderate VI*, *early onset)*.

*“I would love to have kids*, *but I worry*…*about bringing them up”*

*(Female*, *SVI/BL*, *late onset)*.

Within this final stage of *becoming me*, participants described developing and modifying coping strategies which were flexible in nature, and in which they could adapt in the face of vision-specific challenges, meaning that they were able to successfully *rise to challenges*. Having a positive and solution-focused attitude is an important strategy seen throughout childhood and adolescence, but was particularly prominent in later adolescence, as young people developed the cognitive and emotional maturity to overcome challenges imposed by VI.

*“Try*, *try*, *try again and if that fails*, *just try again*! *[…] And after you fail*, *go to bed*!”

*(Male*, *SVI/BL*, *early onset)*.

*“I try to find ways*. *Solve problems […] It’s a problem solving skill*.*”*

*(Male*, *SVI/BL*, *early onset)*.

*“I learnt*, *if I can get a laugh out of it [my impairment]*, *and make other people laugh*, *then why not*?!”

*(Male*, *moderate VI*, *early onset)*.

## Discussion

Our study of young people’s retrospectively collected experience of growing up shows the profound impact of VI, with variations by the characteristics of VI on many aspects of everyday life. We present and describe a snapshot of the potential process whereby a child/young person living with VI (i.e. a childhood-onset, chronic health condition), comes to accept themselves as having a disability, and how this disability will shape their future. Three sequential stages of development emerged from the data as illustrative of children and young people’s experience of normative, age-specific issues, and vision-specific challenges. Just like their sighted counterparts, young people with VI experience dynamic changes and challenges during childhood and adolescence, in the nature of their social relationships; developing independence; and thoughts about the future. These normative aspects of growing up, however, were amplified by the experience of VI and a diverse range of additional, vision-specific, challenges. In this study, aspects of everyday life contributed cumulatively to participants’ developing sense of self, contributing in different ways and at different stages. The themes we developed focused on identity and *becoming me* emerged directly from the data, and best illustrate the process that young people described as important to their experiences of growing-up with VI. It is possible therefore, that beyond this stage, this process is not fixed (i.e. the foundations of identity development may continue to shift during adolescence and into adulthood). Thus, future application of this specific process will require extensive further validation.

Overall, our findings are in keeping with the extant literature in developmental psychology and resonate with some key theories of psychosocial development. For example, Erikson proposed that personality develops in a predetermined order through eight stages of psychosocial development, spanning infancy to adulthood, each involving a psychosocial crisis. According to Erikson, the 5^th^ stage (spanning 12–18 years) is characterized by an individual’s exploration of their sense of self and identity. The frequency and nature in which participants spontaneously emphasized the shaping of their sense of self over time, may be explained using some elements of Erikson’s description of an identity *crisis*. An identity crisis is thought to be “precipitated both by the individuals’ readiness and by society’s pressure” [34] (p.180), and to take place when 4 conditions have been met: a certain level of cognitive development must have been attained [35, 36]; puberty has to have occurred; a certain amount of physical growth must have been recently achieved; and cultural pressures have to be pushing the individual towards an identity re-synthesis [37]. In this study, elements of the identity crisis were most prominent in the final stage within which young visually impaired people began thinking about the future, and were faced with assimilating pressures from society (e.g. to follow a particular career path or start a family) with the restrictions imposed by VI.

Traditionally, young people are thought to resolve the identity crisis through establishing a realistic appraisal of the self, blended out of past, present and future [34, 35, 38]. Ideologies and beliefs which are congruent with the developing sense of self are retained, and those which are incongruent are discarded. Recently published literature, however, suggests that identity development may not follow the same pattern when young people are faced with disability or illness, particularly when onset is late and symptoms fluctuate [39]. Participants in the current study with late onset VI and/or progressively deteriorating vision, similar to those living with late-onset Crohn’s disease and Juvenile Idiopathic Arthritis [39], discussed acceptance of their condition, and efforts to adapt and explore alternative options, as important components of their search for an identity. Thus, it is possible that late-onset disability and/or fluctuating symptoms influence not only the *outcome* of the identity crisis, but also the *process* in which young people integrate their experience into a meaningful representation.

Young people with VI are traditionally thought to be at increased risk of social isolation [40]. However, there are mixed conclusions about the similarities of social support and networks among young people with and without VI. For example, young people with VI have reported lower levels of perceived support from peers than their sighted counterparts [41], while no between-group differences have been reported elsewhere [25, 42]. Dutch young people living with VI have reported lower levels of perceived parental support than their sighted peers [42], whereas a Finnish study found no differences in levels of parental support [25]. Exploring the relationship between *source* of social support and self-esteem, relationships with friends, but not with parents, were protective of low self-esteem [25]. Just like sighted adolescents, young people in the current study described a trajectory in which their social relationships transitioned during childhood, from family-centred, to peers, and eventually towards romantic partners [43, 44]. The nature of functional reward sought from social relationships meant that strong relationships, once established, were considered particularly valuable [45]. Only through the permits of these relationships, participants were able to explore and commit to personal values and ideologies [46], facilitating their developing sense of self. The role of social support from adults, such as parents and teachers, as discussed in the current study, seemingly transitioned in the later stages of adolescence, as young people began to consider their future. These relationships which had been established, and strengthened on the premise of functional support, later became instrumental for orientations towards the future.

The prevalence of mental health conditions among young people in the UK has been recently termed a ‘silent catastrophe’, with the number of adolescents reporting such conditions rising at a rate of fivefold in the last 10 years [47], and thought to peak at the age of 17–19 years [48]. Some literature suggests that adolescents with chronic physical health conditions may be more likely to develop psychiatric and behavioural disorders [49], and be depressed or have low self-esteem [50] than those without a disability. Among young adults living with chronic illness, a strong sense of identity has been associated with less depressive symptoms and better coping strategies [51]. These statistics, combined with the findings from the current study, indicate that the need for clinical monitoring of subjective well-being among adolescents living with chronic conditions such as VI, is critical, particularly since fluctuating clinical characteristics can impact the foundations of identity and sense of self. Participants with late-onset and/or progressively deteriorating VI in the current study were unique in their discussion of profound emotional and cognitive efforts to adapt to and accept VI, and thus might potentially be most vulnerable to the negative psychological impact of VI on developing identity. Future research, however, with larger sample sizes is needed before conclusions can be made about how best to support individuals with varying clinical characteristics.

In light of the ‘silent catastrophe’, emotional and psychological well-being for patients with long-term conditions has been prioritised in the NHS’s *Five Year Forward View* [52], generating a discussion about integrating physical and mental health services [53, 54]. Indeed, addressing the psychological needs of adults living with diabetes has been shown to improve physical health, support relationships between patients and health professionals [55], and there is scope for NHS savings [56]. The NHS Mental Health Implementation Plan proposes specifically, integration of services to support the needs of children and young people with physical health problems, and an ambitious and comprehensive ‘offer’ is expected to transform services by 2023/24 [57]. Whilst this ‘offer’ will take time and resources to develop and refine, it does not stop health professionals who are working closely with young people living with VI from engaging with patients’ subjective experience of VI. This can be done, for example, using robust and developmentally appropriate patient-reported outcome measures of vision-related quality of life [26], which may be particularly valuable when monitoring well-being over time with young people who, as shown in this study, are most at risk of negative psychosocial consequences of VI. The feasibility of combining the specialist skills of psychologists, and others involved in promoting psychological well-being among young people with VI, and clinicians who seek to assess, and support physical functioning, however, is an important focus for future research.

There are many, well-documented challenges associated with conducting interviews with children and young people [58]. For example, young people may strive to conform to their perceptions about the expectations of researchers [59]. For this reason, we carefully planned the interview procedure, and used ice-breaker activities, open-ended questions, and age-appropriate language with fostered a secure environment in which participants felt comfortable and confident to self-report. The rich qualitative data we present, and remarkable transparency and sincerity on behalf of participants, reflects these efforts. We also achieved a robust sample size which contained participants with a range of demographic and clinical characteristics. It is possible, however, that we have captured the attitudes and experiences of young people who are coping particularly well with VI, and are thus, more willing to discuss their experience that those experiencing greater psychological, emotional or functional distress. Although we collected robust data from participants with a range of clinical characteristics, the spread of participants with certain characteristics, such as late-onset VI was skewed, and conclusions regarding variation between participants with different clinical characteristics must be interpreted with caution until further research can be done. Additionally, this study is the result of secondary data analysis with the primary purpose to develop a collection of instruments [26, 27]. Data collected with the primary aim to explore the retrospective experience of growing up with VI may provide more detail.

To conclude, we present a snapshot of the self-reported descriptions of growing up with VI, from the perspectives of young people on the brink of adulthood, which illustrate the potential sensitive nature in which a chronic condition, with fluctuating progression and severity, can influence normative aspects of growing up. Using qualitative methods, we captured young visually impaired people’s perceptions of specific changes in their psychosocial development, adding to the growing body of quantitative literature which highlights the far-reaching impact of VI on psychosocial well-being during childhood and adolescence. The findings we report add granularity to existing research studies focused on identity and self-concept among children living with VI as, when given the opportunity, young people spontaneously described the *process* whereby children come to accept VI as part of their identity. These findings support the value of developing integrated physical and mental health services for this population and highlight the need for long-term monitoring of well-being and mental health in a population who may be particularly vulnerable to challenges and changes in developing a sense of identity.

## Supporting information

S1 File Interview topic guide. (PDF)

## References

[1] Hall GS. Adolescence: Its Psychology and its Relations to Physiology, Anthropology, Sociology, Sex, Crime, Religion and Education. New York: D. Appleton; 1916.

[2] Somerville LH, Jones RM, Casey B. A time of change: behavioral and neural correlates of adolescent sensitivity to appetitive and aversive environmental cues. Brain Cogn, 2020; 72(1):124–33. 10.1016/j.bandc.2009.07.003. PMC2814936 19695759

[3] Susman EJ, Inoff-Germain G, Nottelmann ED, Loriaux DL., Cutler GB Jr, Chrousos GP. Hormones, emotional dispositions, and aggressive attributes in young adolescents. Child Dev, 1987; 5(4):1114–34. 3608660

[4] Blakemore SJ, Choudhury S. Development of the adolescent brain: implications for executive function and social cognition. J Child Psychol Psychiatry, 2006; 47(3–4):296–312. 10.1111/j.1469-7610.2006.01611.x 16492261

[5] Costello EJ, Mustillo S, Erkanli A, Keeler G, Angold A. Prevalence and development of psychiatric disorders in childhood and adolescence. Arch Gen Psychiatry, 2003; 60(8):837–44. 10.1001/archpsyc.60.8.837 12912767

[6] Rahi JS, Cable N. Severe visual impairment and blindness in children in the UK. Lancet. 2003; 362(9393):1359–65. doi: 10.1016/S0140-6736(03)14631-4 14585637

[7] Solebo AL, Rahi J. Epidemiology, aetiology and management of visual impairment in children. Arch Dis Child. 2014; 99(4):375–9. 10.1136/archdischild-2012-301970 24148891

[8] Khadka J, Ryan B, Margrain TH, Woodhouse JM, Davies N. Listening to voices of children with a visual impairment: A focus group study. Brit J Vis Impair. 2012; 30(3):182–96. 10.1177/0264619612453105.

[9] Cochrane G, Lamoureux E, Keeffe J. Defining the content for a new quality of life questionnaire for students with low vision (the Impact of Vision Impairment on Children: IVI_C). Ophthalmic Epidemiol. 2008; 15(2):114–20. 10.1080/09286580701772029 18432495

[10] Fazzi E, Signorini SG, Bomba M, Luparia A, Lanners J, Balottin U. Reach on sound: a key to object permanence in visually impaired children. Early Hum Dev. 2011; 87(4):289–96. 10.1016/j.earlhumdev.2011.01.032 21316874

[11] Sonksen PM, Dale N. Visual impairment in infancy: impact on neurodevelopmental and neurobiological processes. Dev Med Child Neurol. 2002; 44(11):782–91. doi: 10.1017/s0012162201002936 12418621

[12] Bigelow AE. The development of joint attention in blind infants. Dev Psychopathol. 2003;15(2):259–75. https://doi.org/10.1017.S054579403000142 12931827 10.1017/s0954579403000142

[13] Pérez-Pereira M, Conti-Ramsden G. Language Development and Social Interaction in Blind Children. Hove, East Sussex: Psychology Press; 2013.

[14] Hughes M, Dote-Kwan J, Dolendo J. A close look at the cognitive play of preschoolers with visual impairments in the home. Except Child. 1998; 64(4):451–62.

[15] Greenaway R, Dale NJ. Congenital visual impairment. In: Cummings L, editor. Research in Clinical Pragmatics. Perspectives in Pragmatics, Philosophy & Psychology. Volume 11. Cham Switzerland: Springer International Publishing AG; 2017. pp. 441–69. doi: 10.1186/s40064-016-2684-5 27512642 PMC4961664

[16] Huurre TM, Aro HM. Psychosocial development among adolescents with visual impairment. Euro Child Adolesc Psychiatry. 1998; 7(2):73–8. doi: 10.1007/s007870050050 9712373

[17] Huurre TM, Aro HM. The Psychosocial Weil-Being of Finnish Adolescents with Visual Impairments versus those with Chronic Conditions and those with no Disabilities. J Vis Impair Blind. 2000; 94(10):625–37.

[18] Bolat N, Dogangun B, Yavuz M, Demir T, Kayaalp L. Depression and anxiety levels and self-concept characteristics of adolescents with congenital complete visual impairment. Turk Psikiyatri Derg. 2011; 22(2):77–82. 21638229

[19] Lifshitz H, Hen I, Weisse I. Self-concept, adjustment to blindness, and quality of friendship among adolescents with visual impairments. J Vis Impair Blind. 2007; 101(2):96–107.

[20] Kef S. Psychosocial adjustment and the meaning of social support for visually impaired adolescents. J Vis Impair Blind. 2002; 96(1):22–37.

[21] Sacks SZ, Wolffe KE. Lifestyles of adolescents with visual impairments: An ethnographic analysis. J Vis Impair Blind. 1998; 92(1):7–17.

[22] Sacks SZ, Wolffe KE, Tierney D. Lifestyles of students with visual impairments: Preliminary studies of social networks. Except Child. 1998; 64(4):463–78.

[23] Garaigordobil M, Bernarás E. Self-concept, self-esteem, personality traits and psychopathological symptoms in adolescents with and without visual impairment. Span J Psychol. 2009; 12(1):149–60. doi: 10.1017/s1138741600001566 19476228

[24] Augestad LB. Self-concept and self-esteem among children and young adults with visual impairment: A systematic review. Cogent Psychol. 2017; 4(1):1319652. 10.1080/23311908.2017.1319652.

[25] Huurre TM, Komulainen EJ, Aro HM. Social support and self-esteem among adolescents with visual impairments. J Vis Impair Blind. 1999; 93(1):26–37.

[26] Tadić V, Robertson A, Cortina-Borja M, Rahi J, Child VP-ROM. An age-and stage-appropriate patient-reported outcome measure of vision-related quality of life of children and young people with visual impairment. Ophthalmology. 2019;19(3):1964–5. 10.1016/j.ophtha.2019.08.033 31623869

[27] Robertson AO, Tadić V, Cortina-Borja M, Rahi JS. A patient-reported outcome measure of functional vision for children and young people aged 8 to 18 years with visual impairment. Am J Ophthalmol. 2020; 219:141–53. 10.1016/j.ajo.2020.04.021 32360333

[28] World Health Organization. International Statistical Classification of Diseases. 11th Revision. Geneva: World Health Organization; 2018.

[29] Guest G, Bunce A, Johnson L. How many interviews are enough? An experiment with data saturation and variability. Field Methods. 2006; 18(1):59–82. 10.1177/1525822X05279903.

[30] Fusch PI, Ness LR. Are we there yet? Data saturation in qualitative research. Qual Rep. 2015; 20(9):1408.

[31] Castleberry A. NVivo 10 [software program]. Version 10. QSR International. American Journal of Pharmaceutical Education. 2014; 7(1):25. 10.5688/ajpe78125.

[32] Braun V, Clarke V. Thematic analysis. In: Cooper H, Camic P.M, Long D.L, Panter A.T, Rindskopf D, S K.J., editors. APA Handbooks in Psychology APA Handbook of Research Methods in Psychology. Vol 2. Research designs: quantitative, qualitative, neuropsychological, and biological: American Psychological Association; 2012. pp. 57–71.

[33] Smith T, Noble M, Noble S, Wright G, McLennan D, Plunkett E. The English Indices of Deprivation 2015. London: Department for Communities and Local Government; 2015.

[34] Erikson EH. Identity and the Life Cycle: A Reissue. New York: WW Norton; 1980.

[35] Erikson EH. Identity: Youth and Crisis. New York: WW Norton; 1968.

[36] Erikson EH. Life History and the Historical Moment. New York: WW Norton; 1975.

[37] Cote JE, Levine C. A formulation of Erikson’s theory of ego identity formation. Dev Rev. 1987; 7(4):273–325.

[38] Bronfenbrenner U. Ecological perspective of human development. In: Muuss R, editor. Theories of Adolescence. New York: McGraw-Hill; 1996. pp. 312–38. doi: 10.1037//1076-8998.1.2.131

[39] Wicks S, Berger Z, Camic PM. It’s how I am… it’s what I am… it’s a part of who I am: a narrative exploration of the impact of adolescent-onset chronic illness on identity formation in young people. Clin Child Psychol Psychiatry. 2019; 24(1):40–52. 10.1177/1359104518818868 30789046

[40] Huurre TM. Psychosocial Development and Social Support Among Adolescents with Visual Impairment. Finland: Tampere University Press; 2000.

[41] Kef S, Deković M. The role of parental and peer support in adolescents well-being: a comparison of adolescents with and without a visual impairment. J Adolesc. 2004;27(4):453–66. 10.1016/j.adolescence.2003.12.005 15288754

[42] Kef S, Hox JJ, Habekothe H. Social networks of visually impaired and blind adolescents. Structure and effect on well-being. Soc Networks. 2000; 22(1):73–91. 10.1016/S0378-8733(00)00022-8.

[43] Larson R, Richards MH. Daily companionship in late childhood and early adolescence: Changing developmental contexts. Child Dev. 1991; 62(2):284–300. doi: 10.1111/j.1467-8624.1991.tb01531.x 2055123

[44] Steinberg L, Morris AS. Adolescent development. Annu Rev Psychol. 2001; 52(1):83–110. 10.1146/annurev.psych.52.1.83 11148300

[45] Buhrmester D. Need fulfillment, interpersonal competence, and the developmental contexts of early adolescent friendship. In: Bukowski W. M, Newcomb A. F, Hartup W.W., editors. Cambridge Studies in Social and Emotional Development The Company They Keep: Friendships in Childhood and Adolescence, Cambridge University Press; 1998. pp. 158–85.

[46] Kroger J. Identity in Adolescence. 3 ed. East Sussex: Routledge; 2004.

[47] Gunnell D, Kidger J, Elvidge H. Adolescent Mental Health in Crisis. Br Med J. 2018; 2608:k2608. doi: 10.1136/bmj.k2608 . 29921659

[48] NHS Digital. Mental Health of Children and Young People in England, 2017. https://digital.nhs.uk/data-and-information/publications/statistical/mental-health-of-children-and-young-people-in-england/2017/2017; Sept 2018.

[49] LeBlanc LA, Goldsmith T, Patel DR. Behavioral aspects of chronic illness in children and adolescents. Pediatr Clin North Am. 2003; 50(4):859–78. doi: 10.1016/s0031-3955(03)00072-5 12964698

[50] Seigel WM, Golden NH, Gough JW, Lashley MS, Sacker IM. Depression, self-esteem, and life events in adolescents with chronic diseases. J Adolesc Health Care. 1990; 11(6):501–4. 10.1016/0197-0070(90)90110-N 2262397

[51] Luyckx K, Seiffge-Krenke I, Schwartz SJ, Goossens L, Weets I, Hendrieckx C, et al. Identity development, coping, and adjustment in emerging adults with a chronic illness: The sample case of type 1 diabetes. J Adolesc Health. 2008; 43(5):451–8. 10.1016/j.jadohealth.2008.04.005 18848673

[52] NHS England. Five Year Forward View. Leeds, UK: NHS England; 2014.

[53] Naylor C, Das P, Ross S, Honeyman M, Thompson J, Gilburt H. Bringing Together Physical and Mental Health: a New Frontier for Integrated Care. London, UK: The King’s Fund; 2016. https://www.kingsfund.org.uk/publications/physical-and-mental-health.

[54] NHS Confederation. Investing in Emotional and Psychological Wellbeing for Patients with Long-Term Conditions. London, UK: NHS Confederation Mental Health Network; 2012.

[55] Alam R, Sturt J, Lall R, Winkley K. An updated meta-analysis to assess the effectiveness of psychological interventions delivered by psychological specialists and generalist clinicians on glycaemic control and on psychological status. Patient Educ Couns. 2009; 75(1):25–36. 10.1016/j.pec.2008.08.026 19084368

[56] Department of Health. Mental Health Promotion and Mental Illness Prevention: The Economic Case. London, UK: Department of Health; 2011.

[57] NHS England. NHS Mental Health Implementation Plan 2019/20–2023/24. London, UK: NHS England; 2019.

[58] Irwin LG, Johnson J. Interviewing young children: Explicating our practices and dilemmas. Qual Health Res. 2005; 15(6):821–31. 10.1177/1049732304273862 15961878

[59] Weber LR, Miracle A, Skehan T. Interviewing early adolescents: some methodological considerations. Hum Organ. 1994; 53(1):42–7.

