# Supplementary material for: This is me: A qualitative investigation of young people’s experience of growing up with visual impairment
Source: PLoS One. 2021 Jul 7;16(7):e0254009. doi: 10.1371/journal.pone.0254009 (PMC8263264; doi:10.1371/journal.pone.0254009)
Supplement: S1 File — (PDF) [file pone.0254009.s001.pdf]

S1 File. Topic guide for interviews with young people aged 16-19

*Topic guide used as part of the broader research programme focused on developing content for an outcome measure capturing vision-related quality of life. Breadth of topics/questions reflects the nature of the outcome measure.

- **Home life**

So firstly, let's start with your home life.

For instance, let's start with your family? Who do you live with?

Probes:<br>Do you have any siblings?<br>How do you get on with them?<br>What do you like to do with them?<br><br><br>Have you always lived together?<br>Have you always lived in the same house?<br>Does anyone else in your family have problems with their eyes?

Can you tell me about the sorts of things you do when you're at home?

Probes:<br>What did you do when you were at home yesterday/at the weekend?<br>- did you enjoy doing that?<br>- did you find that difficult?<br>- did you need help with that?<br>Do you have any pets?<br>Do you have your own bedroom at home?<br>- who do you share it with?<br>- do you mind sharing?

Is there anything in particular you find difficult doing at home because of your eyesight?

Probes:<br>What kind of things?<br>How about finding your way around the house?<br>Do you need help getting ready in the morning?<br>- who helps you?<br>Does anyone do your hair/make-up for you?<br>How about your sleep?<br>- why?

How do you feel about that?

Independence at home? (only if they've mentioned something)

- you said your parents help you with …?
- do you mind if we talk a little bit more about that in terms of your independence?

- **School**

Let's move on to talking about your school life now.

Do you go to school or college or University at the moment?

Probes:
If **yes**:

- what kind of school is it? Is there a VI unit?
- are there boys and girls at school?

What sort of things do you like/enjoy about school?

What things do you not like about school?

Probes for both of the above:
Can you describe to me what you did at school yesterday? Is that a typical day for you?
The classrooms, lessons, favourite subject, teachers
What do you like to do at break time/lunchtime?
Who do you spend time with at school?
What do you like doing with your friends/mates?
Do you travel to school by yourself?
What is your favourite subject?

- what subjects are you taking for your GCSE/A levels?
- what do you like so much about…?
- do you have many/how do you feel about your GCSE/A level exams?

Do you have a favourite teacher?

- why?

Do you have a teaching assistant?

- do you like him/her?
- why?

What special equipment, if you use any, do you use at school?

- do you mind using it?

Do you feel your eyesight gets in the way of things you do at school?

Probes:
How does that make you feel?

Is there anything you would like to do at school but can't because of your eyes?

Transition:

Probes:
Have you ever experienced a mainstream/special school?
- which one do you prefer?
- what was different about mainstream/special school?
- how did things change?

Do you think your life at school in general has changed as you've got older?
- did you notice any big changes?
- how did that make you feel?
- how did things change?

Would you like to go to college/university in the future?

Probes:
If **Yes:**
- what subject would you like to study?
- why?
- have you thought about which University you'd like to go to?
- why?

If **No:**
- when did you leave school?
- why did you decide not to go to college?
- have you thought about going back to school/college in the future?

Retrospective (for **yes** and **no**)

How did things change for you when you left/changed school?
How did you feel about changing schools?
Did you mind leaving your friends and teachers at your old school?
Do you remember moving from primary to secondary school?
- what do you remember?
- how did you feel?

- **Leisure life**

Now let's talk a little bit more about your free time.

What do you like to do in your free time?
What activities do you enjoy?

Probes:
What do you enjoy about…?
- where do you go?
- how often do you…?
- who do you do… with?

Do you enjoy exercise?
- how often do you exercise?

Are there any activities that you would like to do but can't because of your eyes?
- how does this make you feel?

Probes:
Do you use any vision devices/technology to help you when you do things outside the house?
- do you like using them?
- do you find it helpful?

Do you enjoy shopping? Do you usually go with your mates or by yourself?
- do you have any difficulties when you go shopping because of your eyes?
- are you able to read the labels and prices of things?
- how about using money?
- do you ever go alone?
- which do you prefer?
- how do you feel about … coming with you?

How about travelling places?
- do you ever do it by yourself?
- do you ever use the bus or train to get to…?
- how does that make you feel?

So in your free time you like to… Could you tell me a bit more about… ?

So what would be the main way you feel your eyesight affects what you can or can't do in your free time?

Is there anything else important about your free time that I haven't asked?

- **Independence**

Let's move on to talking a bit more about independence.

Probes:
Do you have any chores/jobs that you have to do when you're at home?
- do you have any particular responsibilities when you're at home?
- do you enjoy doing…?

Do you ever go outside on your own?
- do you prefer going out on your own or with somebody?

How about privacy, is that OK for you at home?
Do you feel as though you have enough time for yourself when you're at home?

(only if they've mentioned lack of privacy):
Do you have somewhere to go where you know you won't be interrupted/bothered by anyone?
Do you find it hard to get any privacy?

Do you like living at home?
- why [not]?

So at home you like to…

Probes:
- How do you feel about…?
- Can you tell me more about…?

Is there anything you would like to do at home, but can't because of your eyes? How do you feel about that?

Transition

How do you feel your level of independence has changed since you were in primary school/secondary school/ college?

Probes:
Can you remember…?
For example, do you go out on your own now?
What changed for you?
Can you remember a time when things were different?

- **Social Life**

Let's talk a little bit about your social life.

Probes:
Do you have a best friend or group of best friends?
- do they go to the same school/college as you?
- how did you meet?

Where do you like to go/do with your best friends?
- who do you hang out with the most?

Do you and your friends like to wear the same clothes/ follow the same trends? (touches upon appearance)
- why?

Are all you friends girls/boys? (choose same gender to then move on to boyfriends/girlfriends)

Do any of your friends have boy/girlfriends?
* what about you?
* anyone special?
Probe sensitively.

Yes:
- what are they like/can you tell me a bit more about him/her?
- how does that make you feel?
- how/when did you meet?
- what do you like to do together?
- what do your parents think about…?

No:
- have you ever thought about going on a date/out with a boy/girl?
- would you like to?
- do any of your friends have boy/girlfriends?
- how does that make you feel?

Do any of your friends have VI?

Is there anything you find difficult to do with your friends because of your eyes?

Probes:
Is there anything they can do but you can't because of your eyes?
Does that bother you?
Do you spend more time with your friends or family?

- ***Fitting in, being part of the crowd, treated the same***

Do you feel your friends are generally understanding?
- do they understand what it's like for your?
- how does that make you feel?

Transition:

Do you feel like your friendship with … / friendships have changed at all as you've got older?

Probes:
What about when you moved from primary to secondary school? Secondary school to college?
When you moved from mainstream to specialist school? (depending on what they've already told me).
How has that changed as you've got older?

- **Future life/aspirations**

Let's talk a little bit about the future.

Probes:
Have you thought about moving away from home in the future?
- how do you feel about that?

Have you thought about what kind of job you'd like to do in the future?
- what job?
- why?

Have you thought of any things you would like to do in the future, but might not be able to do because of your eyes?

Probes:
Do you have any concerns/worries about the future?

Would you like to travel abroad in the future?

How do you feel about that?

- **Clinical Care**

The last thing I'd like to ask you about is the clinical care that you receive for your eyesight.

Do you still regularly attend clinics?

- where/ which hospital?
- how often do you have to go?

Probes:
How do you find it?

Is there anything about those clinics in particular that bothers you?

Have you ever had to change consultants?

- how did you find that?

Transition:

Are you still seen in the clinics for children or have you transferred to adult care?

Probes: How did you find that process?
Did that bother you?
Would you have done it differently?

End of interview.
